# Supplementary material for: Promiscuous Lipase-Catalyzed Markovnikov Addition of H-Phosphites to Vinyl Esters for the Synthesis of Cytotoxic α-Acyloxy Phosphonate Derivatives
Source: Materials (Basel). 2022 Mar 7;15(5):1975. doi: 10.3390/ma15051975 (PMC8912074; doi:10.3390/ma15051975)
Supplement: Supplementary file 1 [file materials-15-01975-s001.zip › materials-1603179-supplementary.pdf]

materials

Supplementary Materials

Promiscuous Lipase-Catalyzed Markovnikov Addition of H-Phosphites to Vinyl Esters for the Synthesis of Cytotoxic α-Acyloxy Phosphonate Derivatives

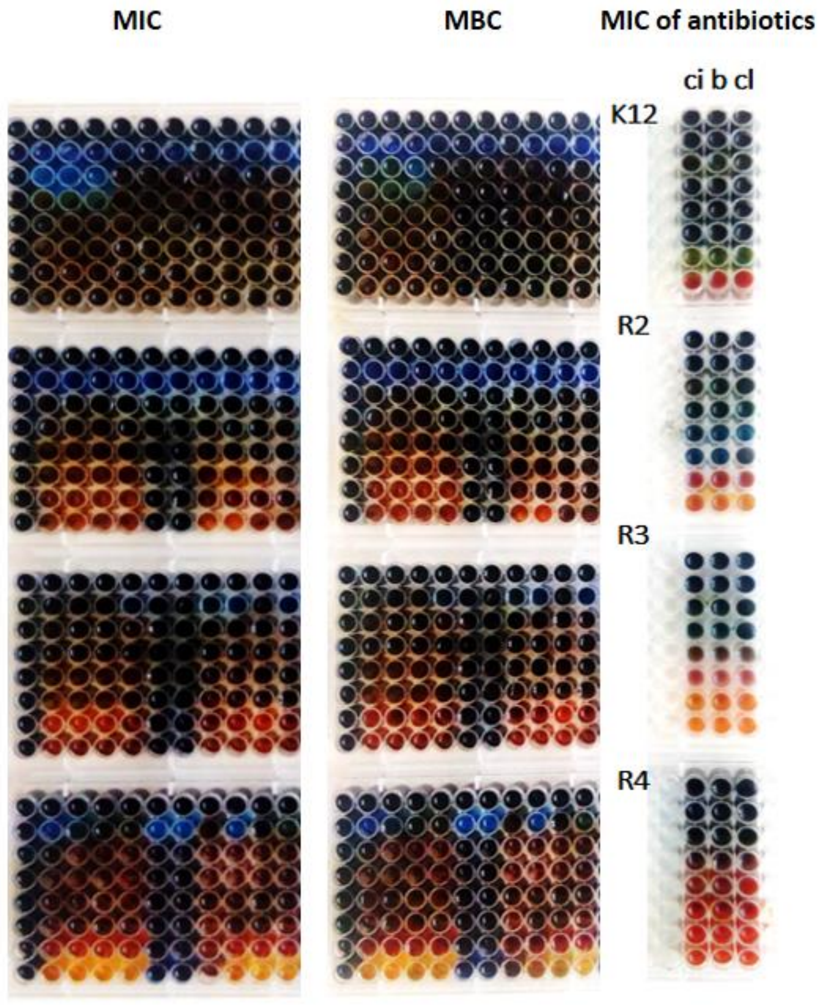

Figure S1. Examples of MIC and MBC on microplates with different concentration of studied compounds (μg/mL^{-1}). Resazurin was added as an indicator of microbial growth with K12, R2, R3, and R4 strains with tested 10 compounds, as described in Table 2. Additionally, examples of MIC with different strains K12, R2, R3, and R4 of studied antibiotics with ciprofloxacin (ci), bleomycin (b), and cloxacillin (cl) in (μg/mL^{-1}).

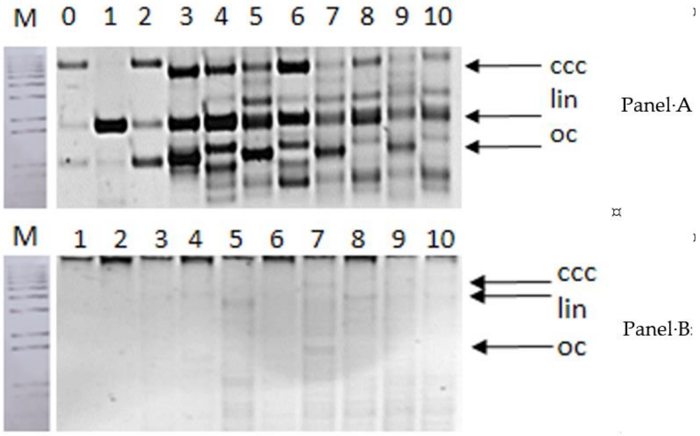

Figure S2. An example of an agarose gel electrophoresis separation of isolated plasmids DNA on R4 strains modified with selected coumarin derivatives (Panel A) from 10 selected compounds, as shown in Figure 3, and digested with repair Fpg protein (Panel B). M = marker.

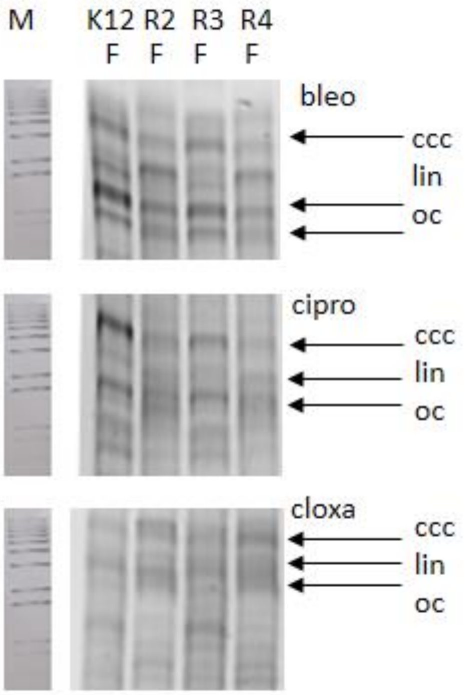

Figure S3. Example of an agarose gel electrophoresis separation of isolated plasmids DNA from K12 and R4 strains modified with antibiotics: bleomycin, ciprofloxacin, and cloxacillin digested (or not) with repair enzymes Fpg. M = marker.

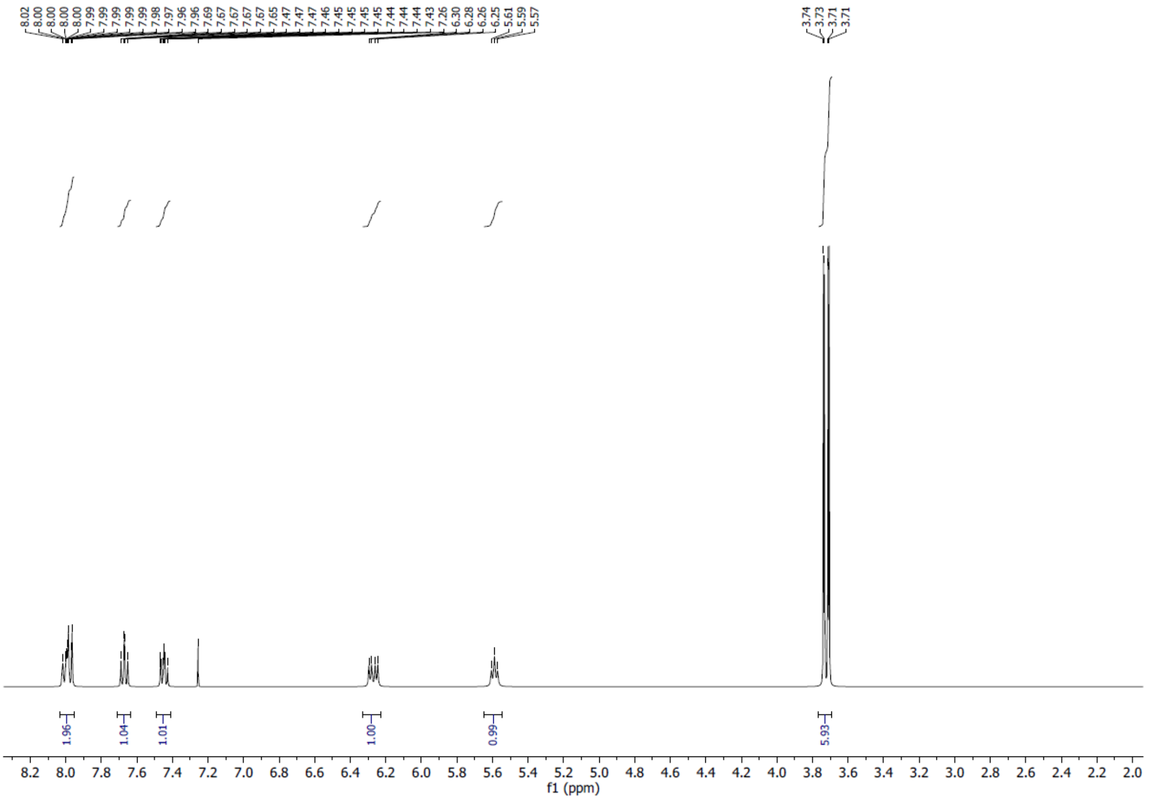

Figure S4. ^{1}H NMR (400 MHz, $CDCl_3$) of dimethyl hydroxy-(2-nitrophenyl)methylphosphonate (**2**).

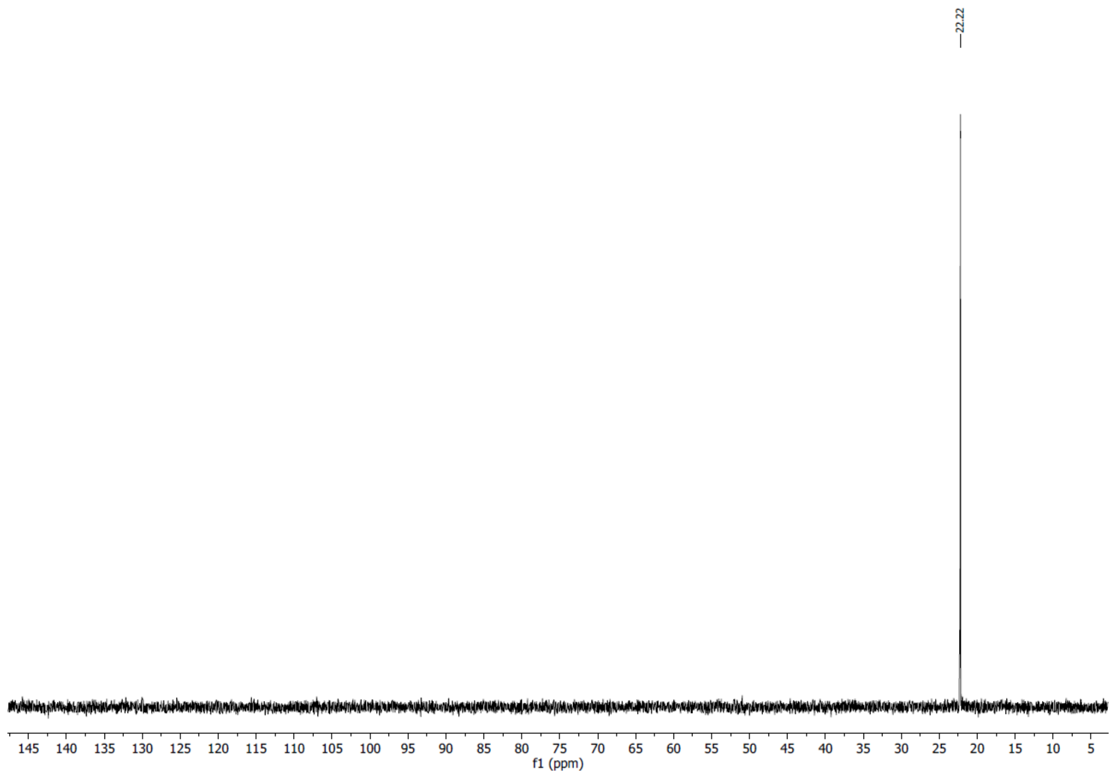

Figure S5. ^{31}P NMR (162 MHz, $CDCl_3$) of dimethyl hydroxy-(2-nitrophenyl)methylphosphonate (**2**).

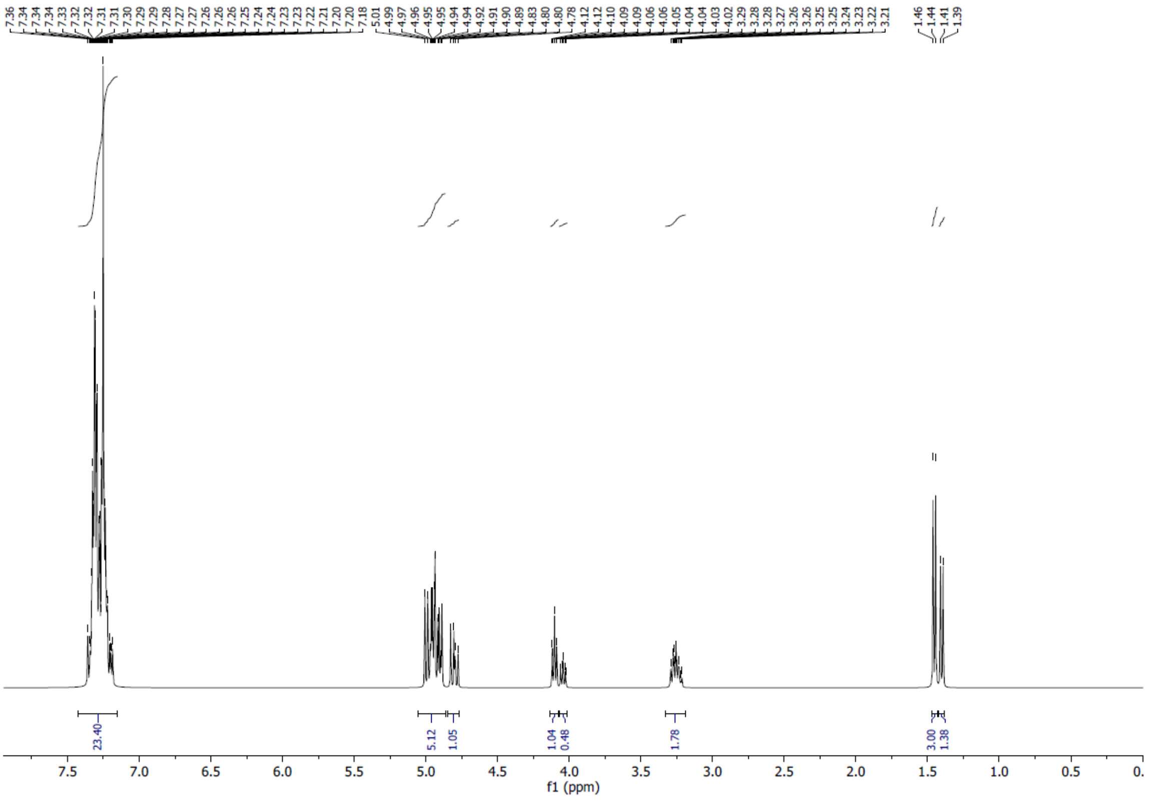

Figure S6. ^{1}H NMR (400 MHz, $CDCl_3$) of dibenzyl (1-hydroxy-2-phenylpropyl)phosphonate (**4**).

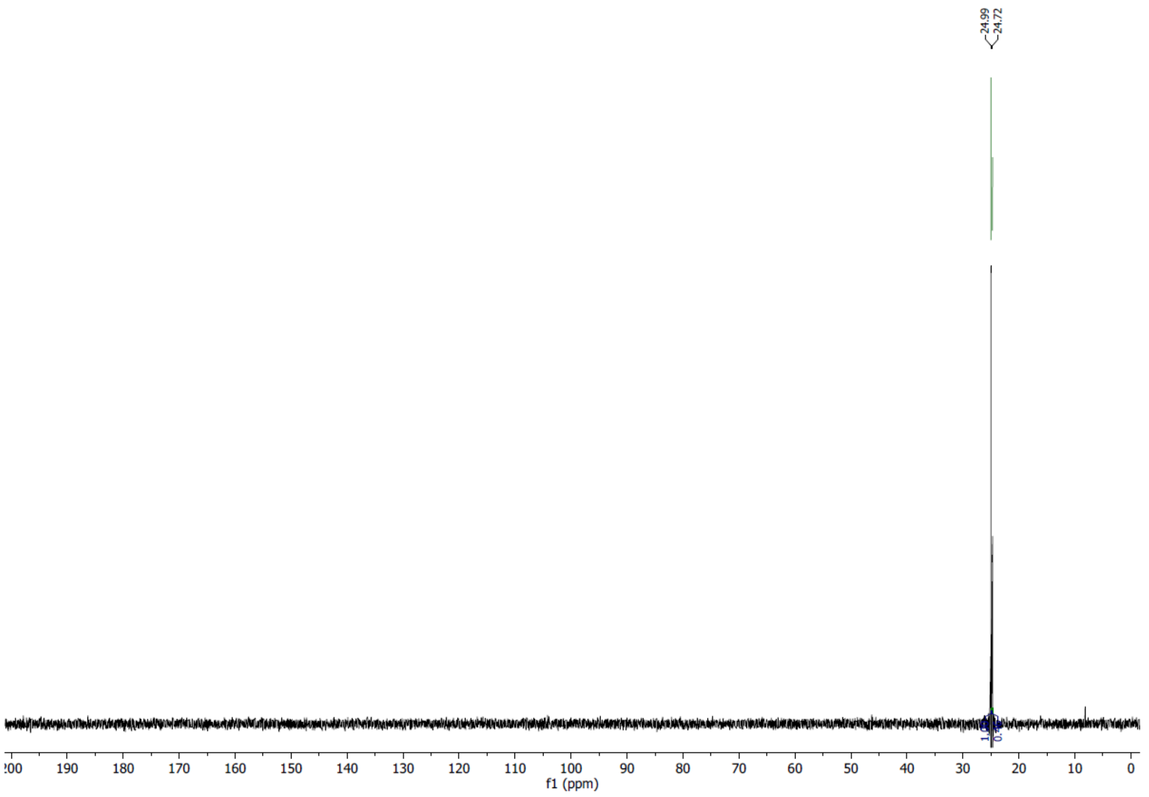

Figure S7. ^{31}P NMR (162 MHz, $CDCl_3$) of dibenzyl (1-hydroxy-2-phenylpropyl)phosphonate (**4**).

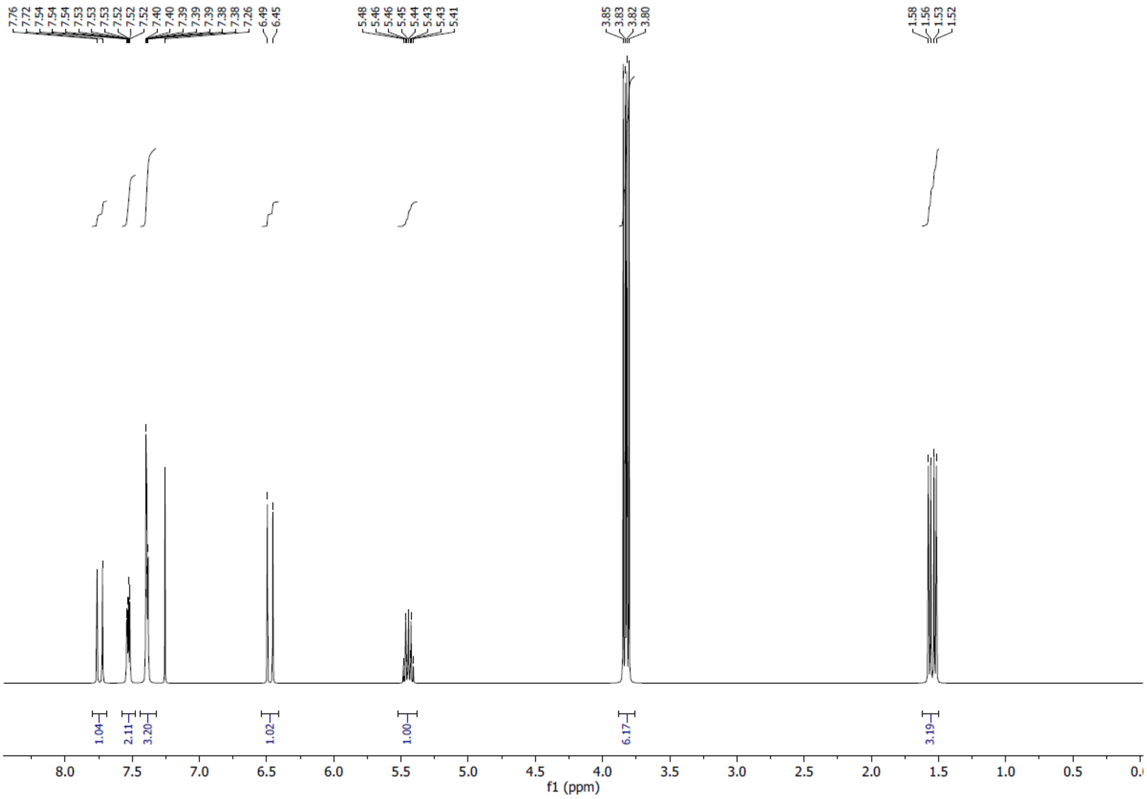

Figure S8. ^{1}H NMR (400 MHz, $CDCl_3$) of 1-(dimethoxyphosphoryl)ethyl cinnamate (**5**).

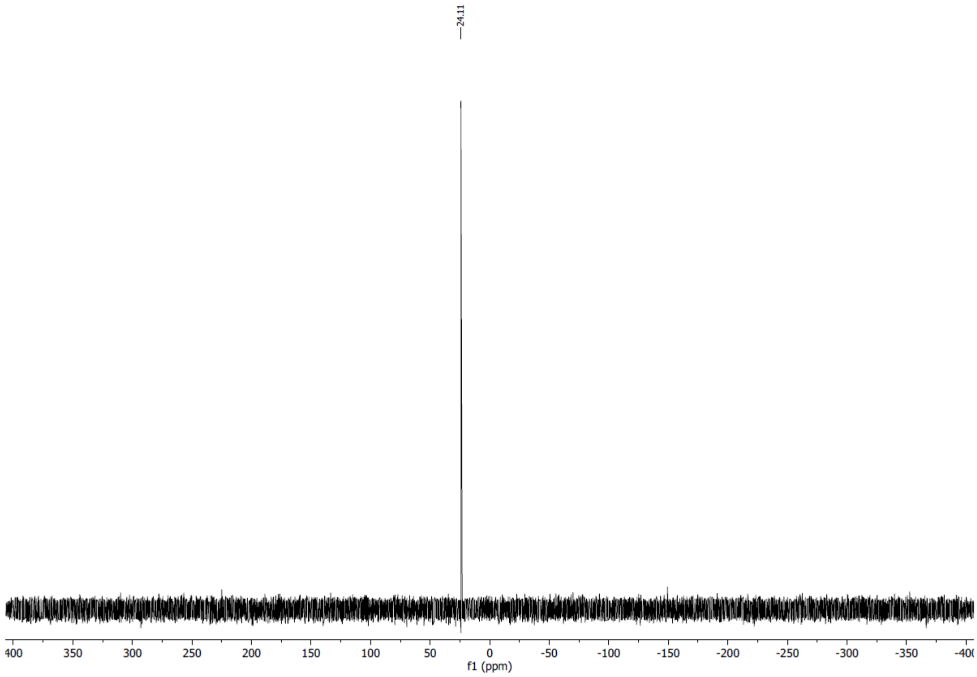

Figure S9. ^{31}P NMR (162 MHz, $CDCl_3$) of 1-(dimethoxyphosphoryl)ethyl cinnamate (**5**).

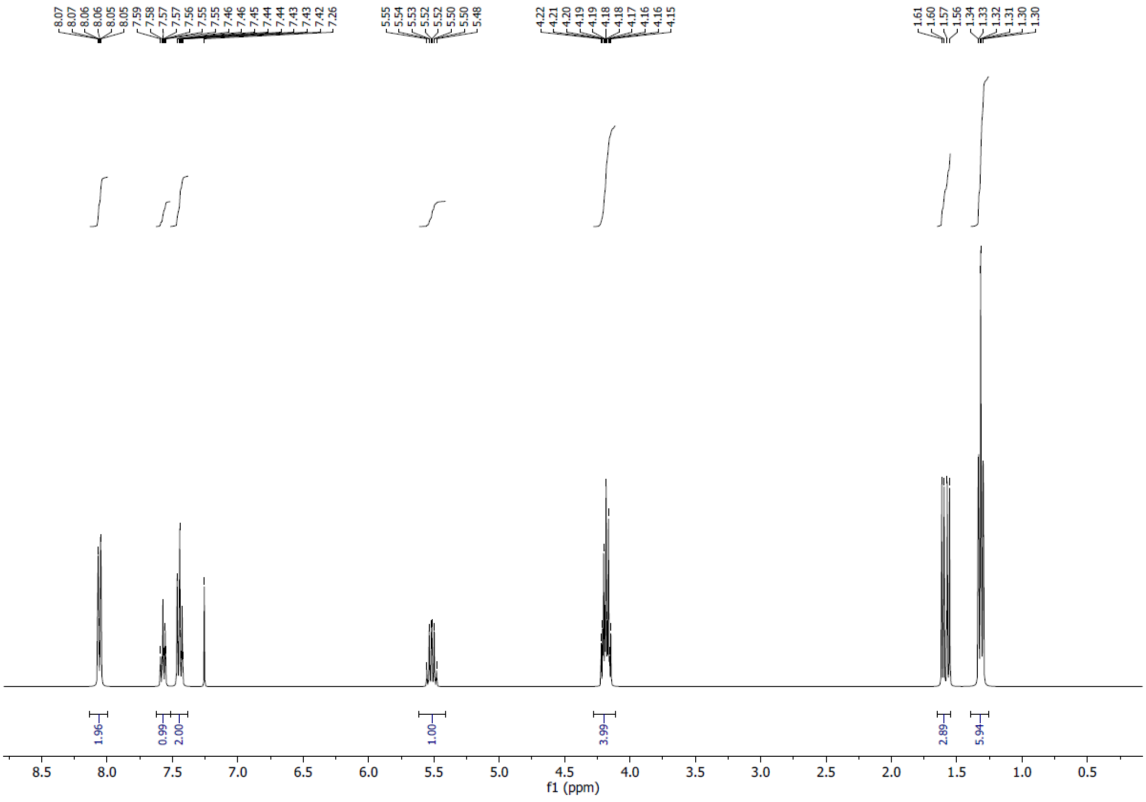

Figure S10. ^{1}H NMR (400 MHz, $CDCl_3$) of 1-benzoyloxy-1-diethylphosphonylethane (**6**).

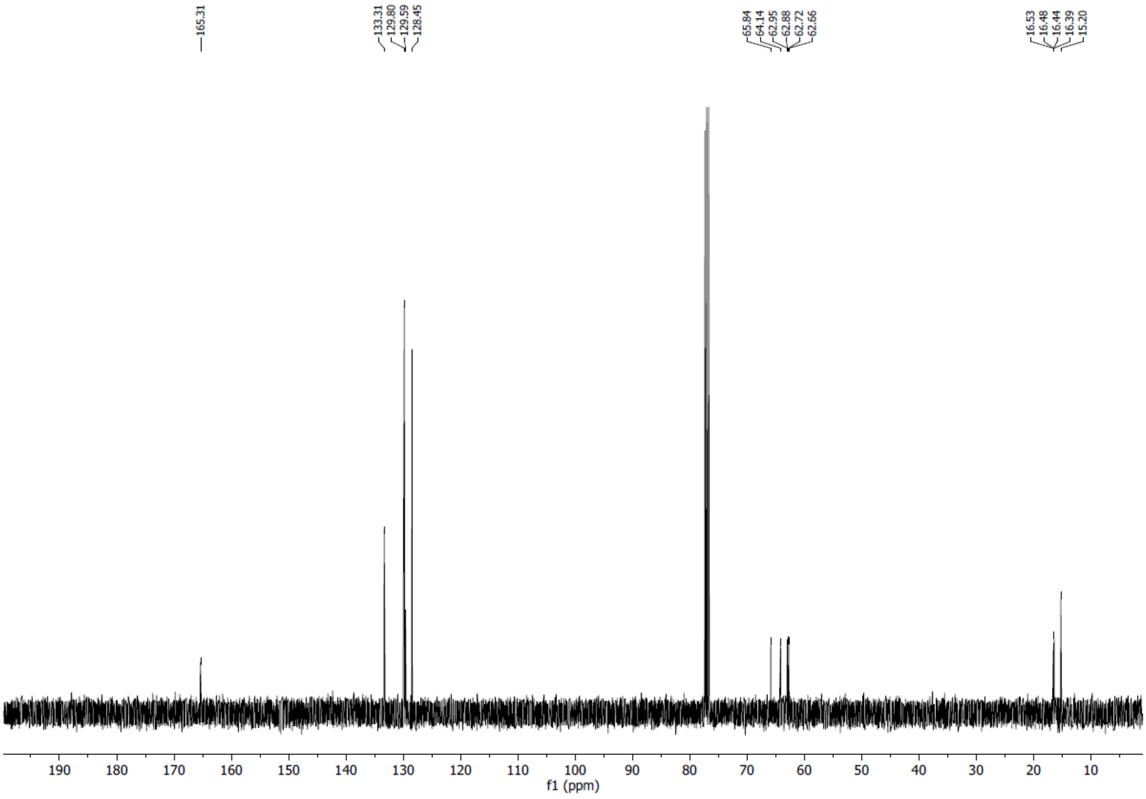

Figure S11. ^{13}C NMR (100 MHz, $CDCl_3$) of 1-benzoyloxy-1-diethylphosphonylethane (**6**).

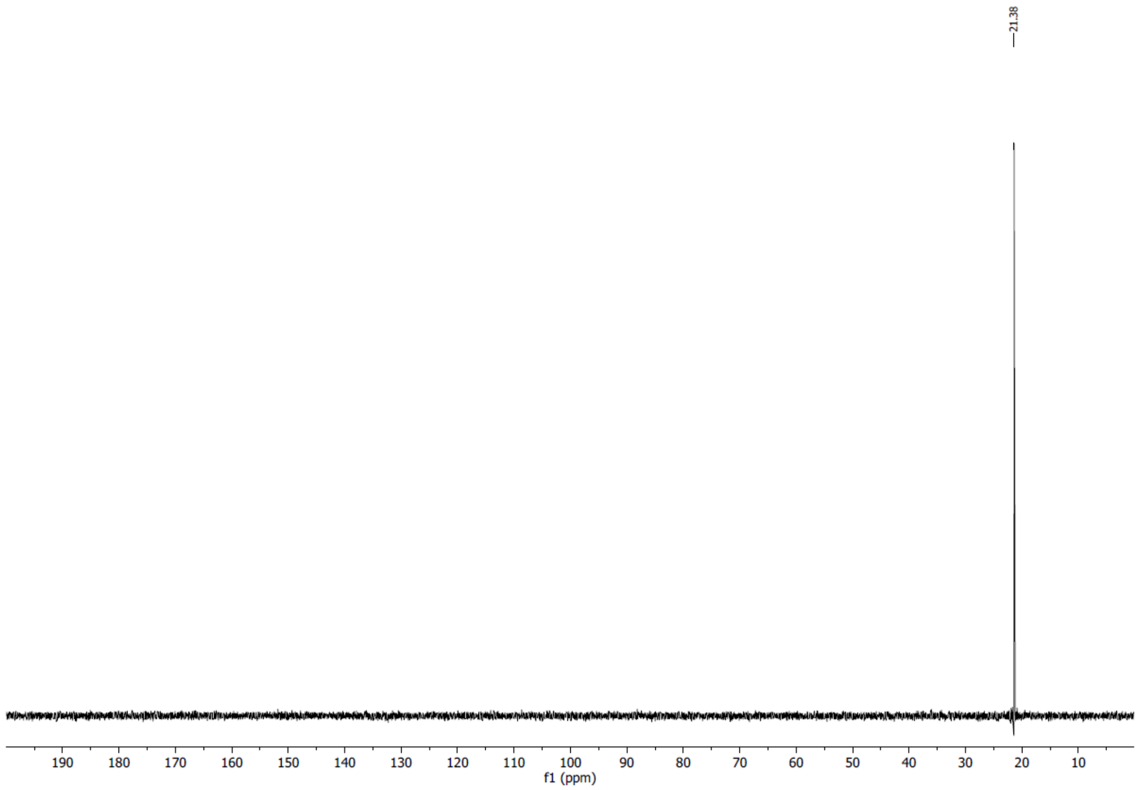

Figure S12. ^{31}P NMR (162 MHz, $CDCl_3$) of 1-benzoyloxy-1-diethylphosphonylethane (**6**).

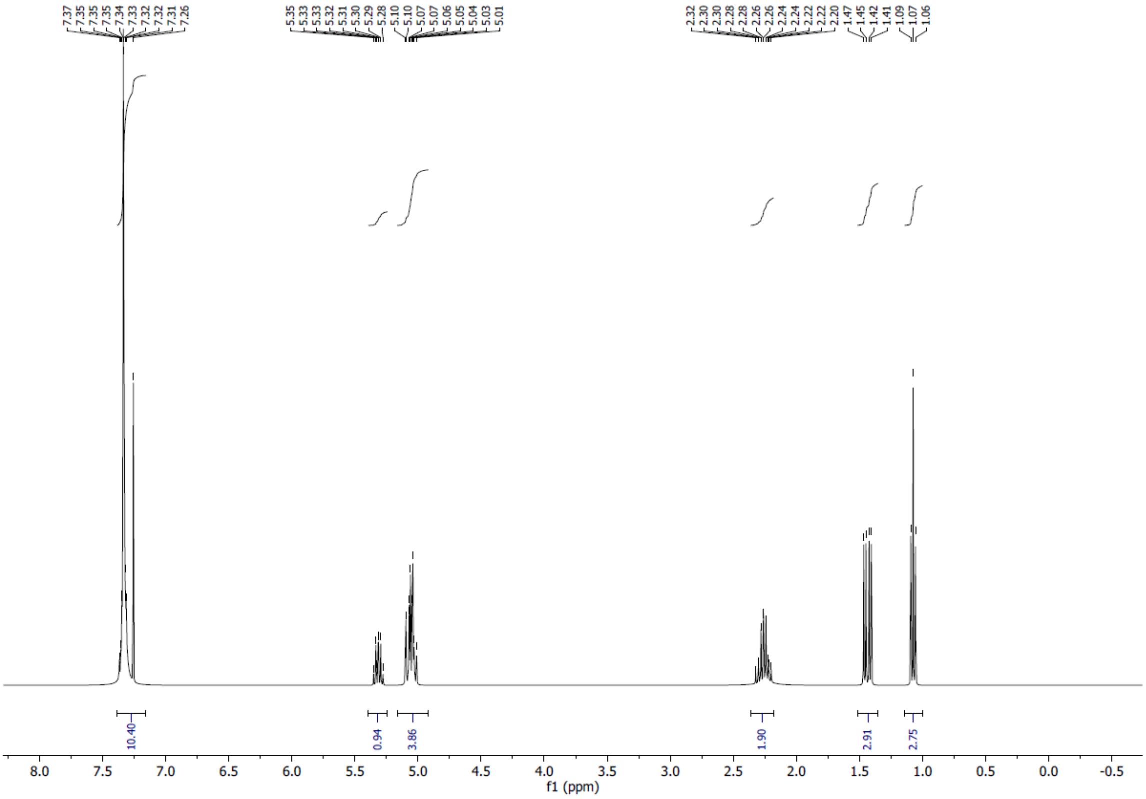

Figure S13. ^{1}H NMR (400 MHz, $CDCl_3$) of dibenzyl(1-propionoxy ethyl)phosphonat (**7**).

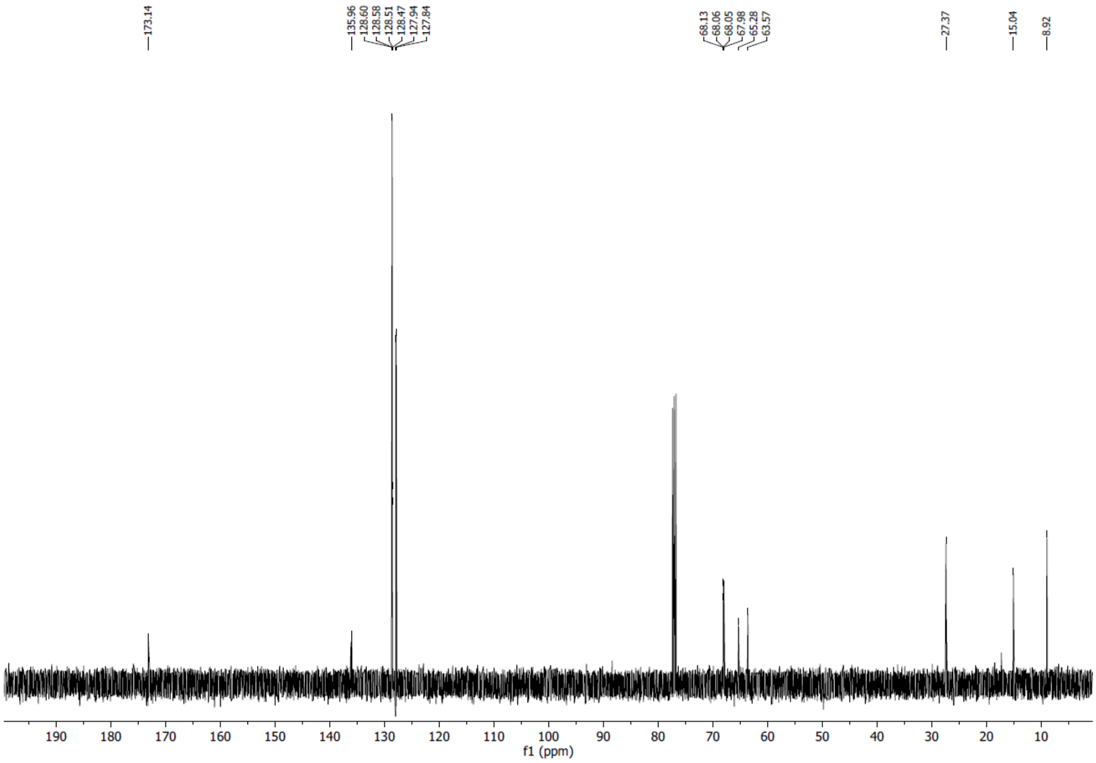

Figure S14. ^{13}C NMR (100 MHz, $CDCl_3$) of dibenzyl(1-propionoxy ethyl)phosphonat (**7**).

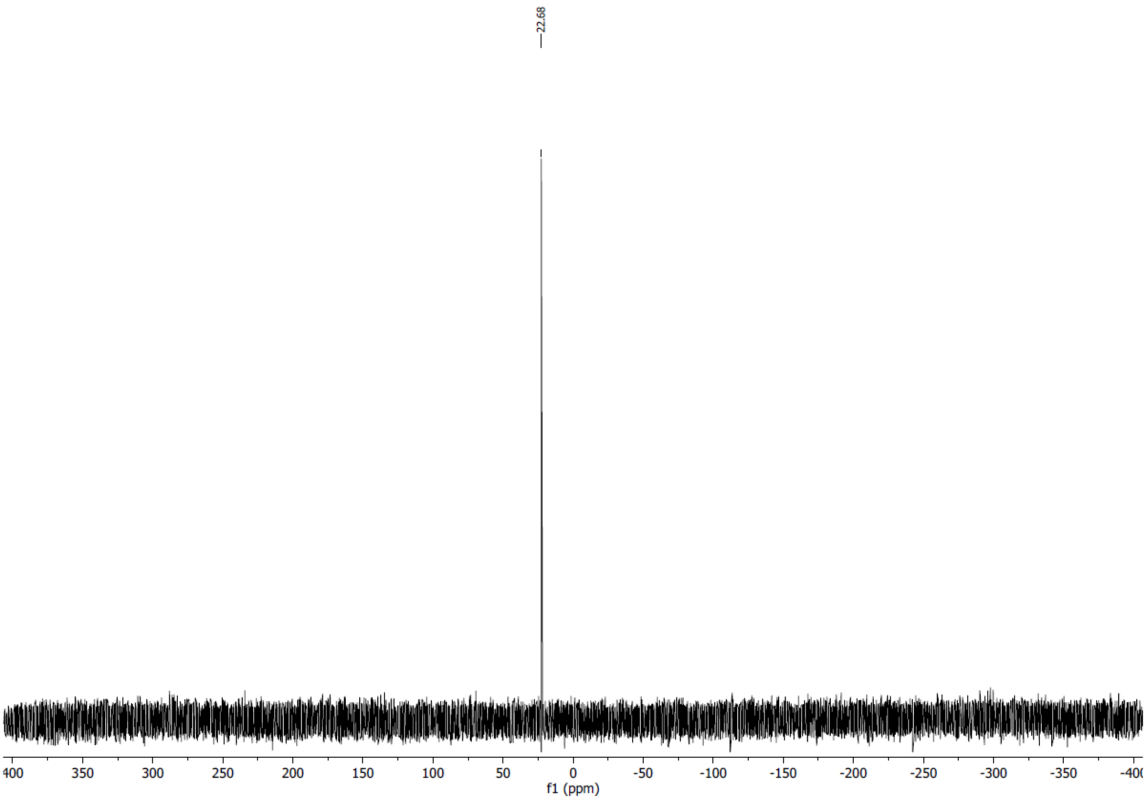

Figure S15. ^{31}P NMR (162 MHz, $CDCl_3$) of dibenzyl(1-propionoxy ethyl)phosphonat (**7**).

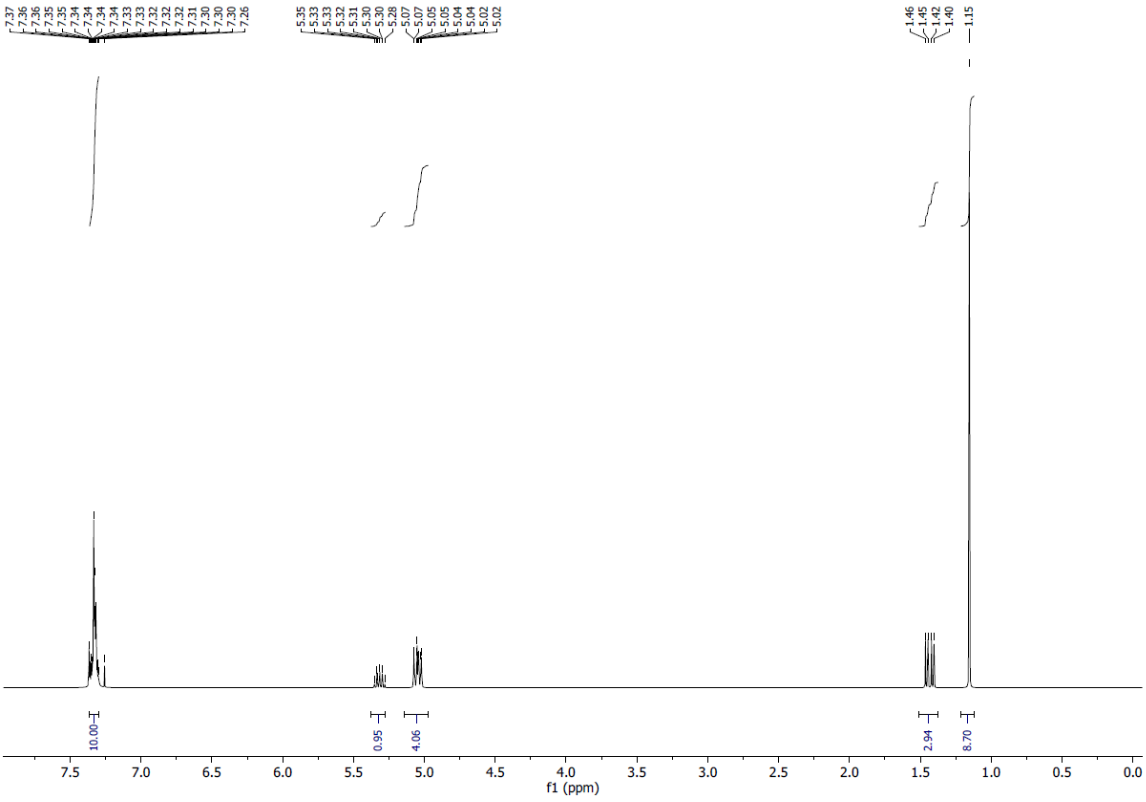

Figure S16. ^{1}H NMR (400 MHz, $CDCl_3$) of 2,2-dimethylpropanyloxy1-dibenzylphosphonylethane (**8**).

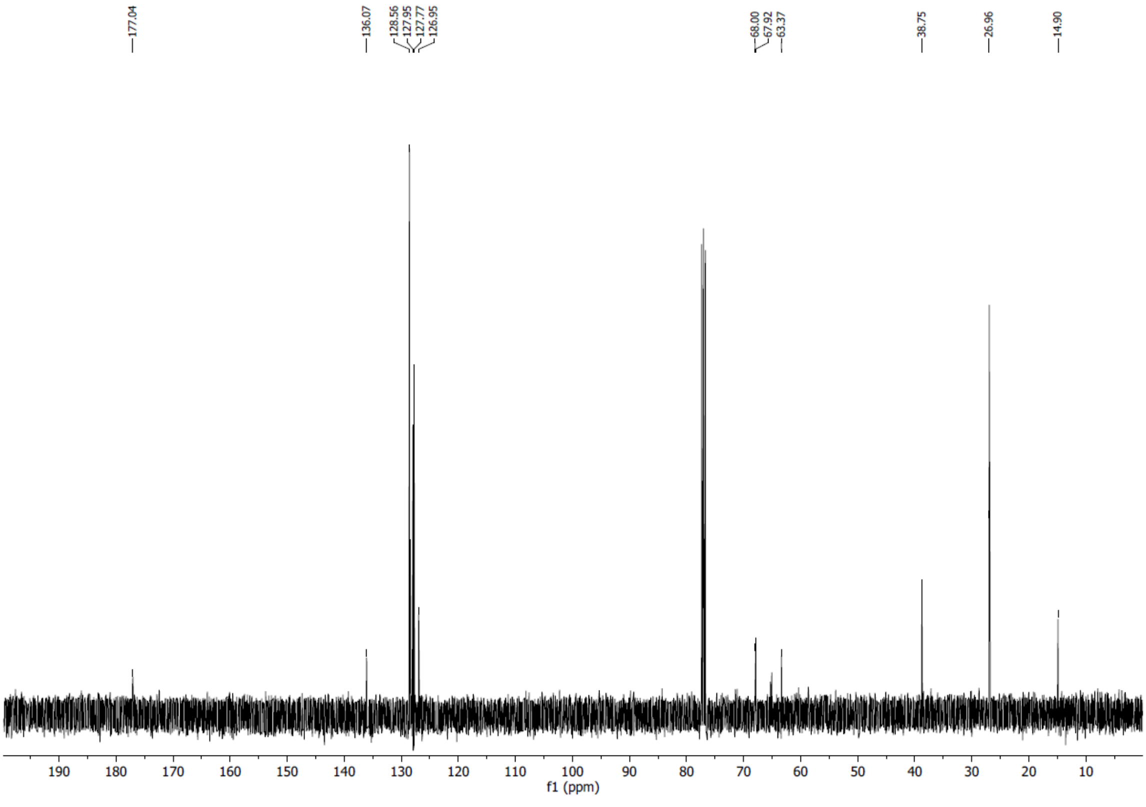

Figure S17. ^{13}C NMR (100 MHz, $CDCl_3$) of 2,2-dimethylpropanyloxy1-dibenzylphosphonylethane (**8**).

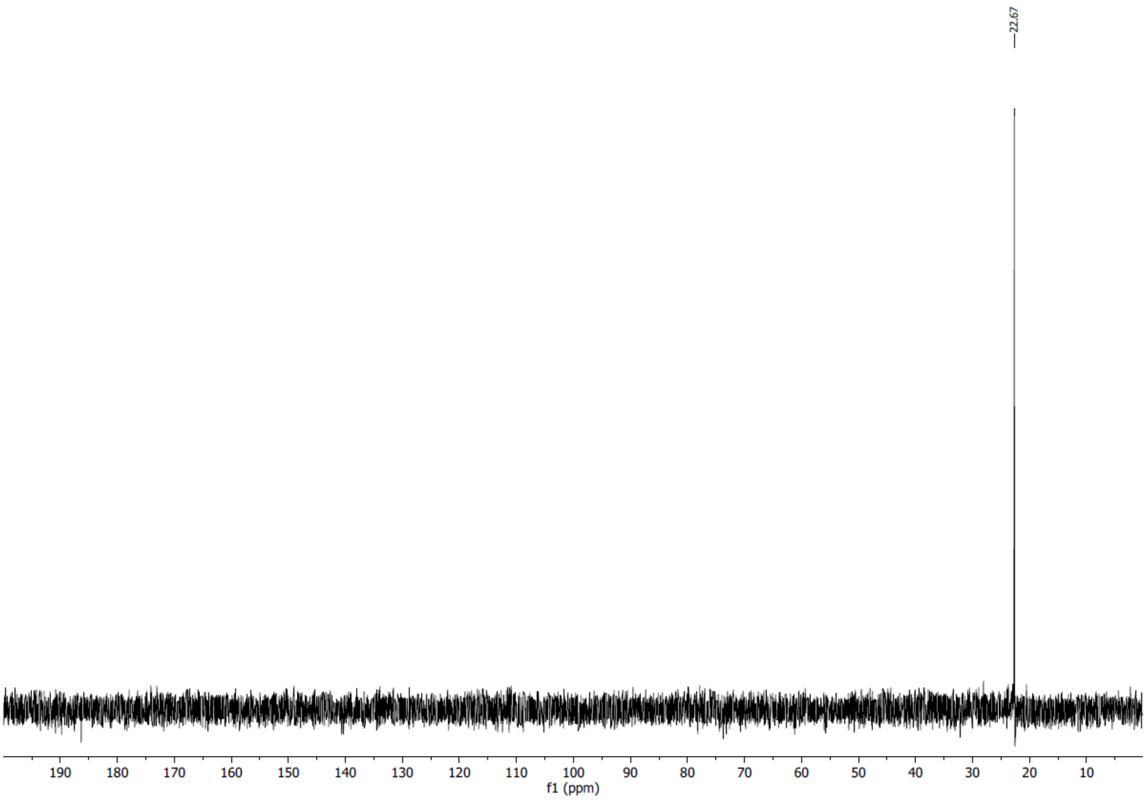

Figure S18. ^{31}P NMR (162 MHz, $CDCl_3$) of 2,2-dimethylpropanyloxy1-dibenzylphosphonylethane (**8**).

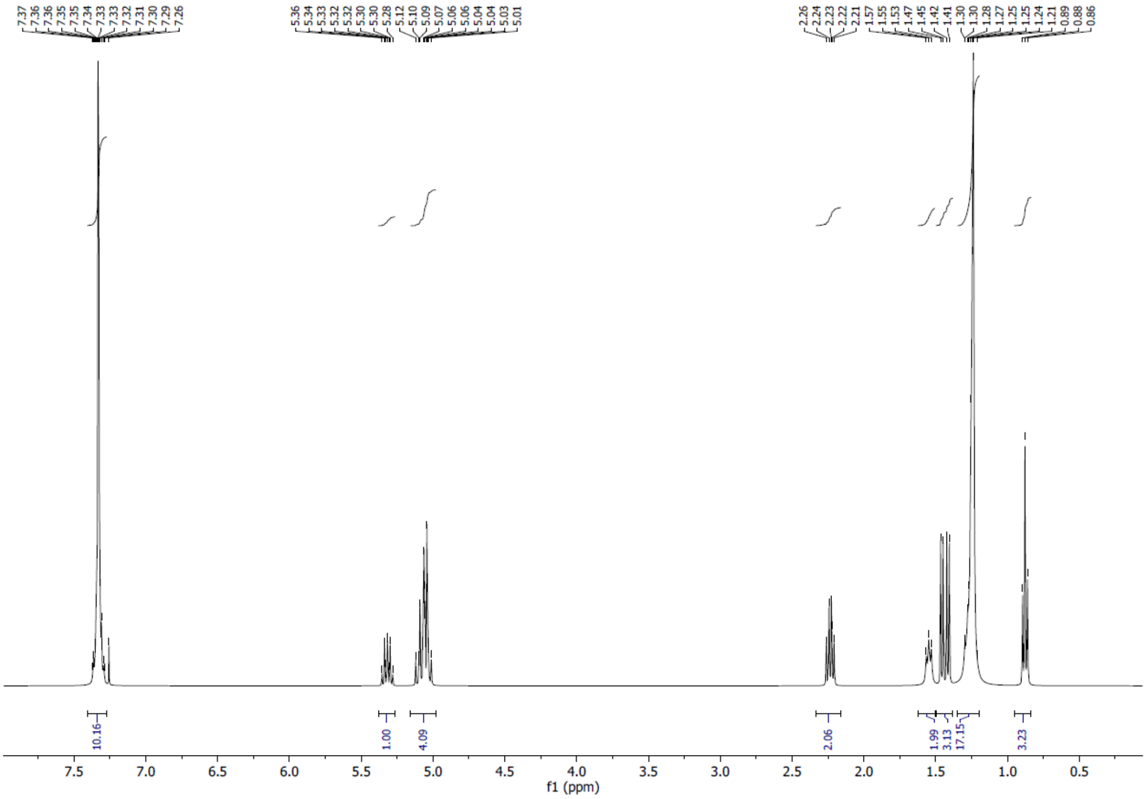

Figure S19. ^{1}H NMR (400 MHz, $CDCl_3$) of 1-(dibenzylphosphoryl)ethyl dodecanoate (**9**).

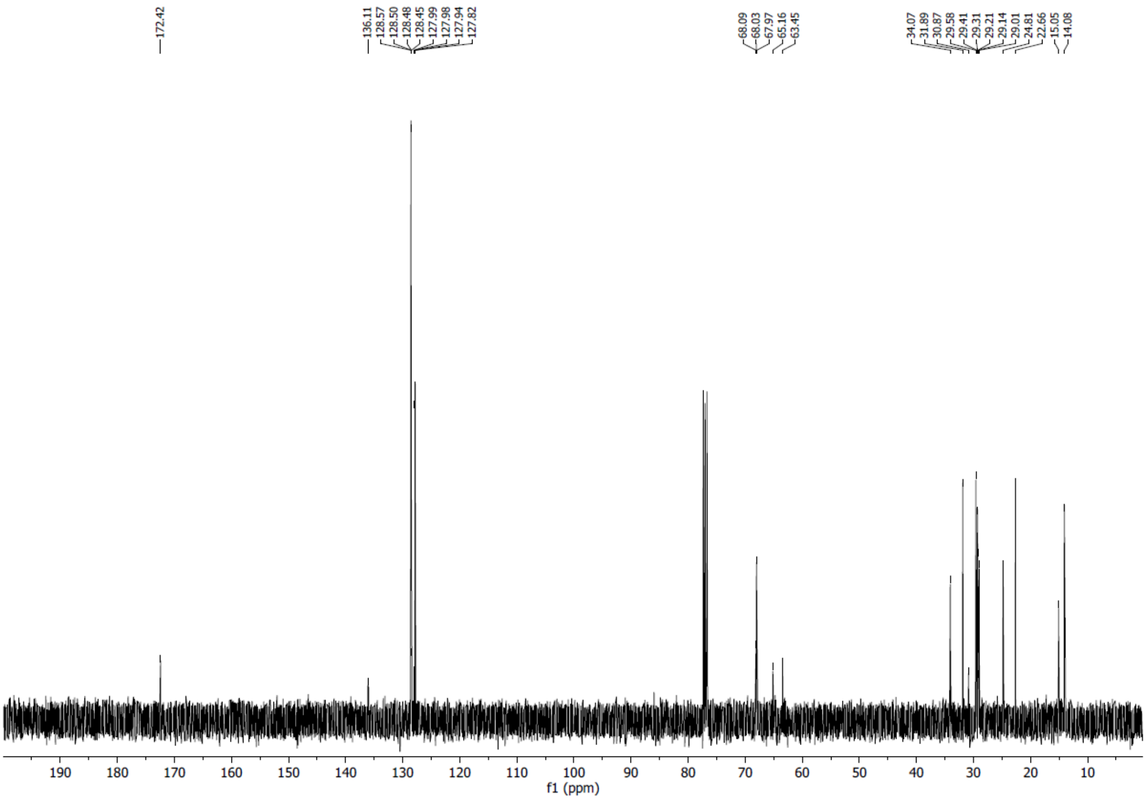

Figure S20. ^{1}H NMR (100 MHz, $CDCl_3$) of 1-(dibenzylphosphoryl)ethyl dodecanoate (**9**).

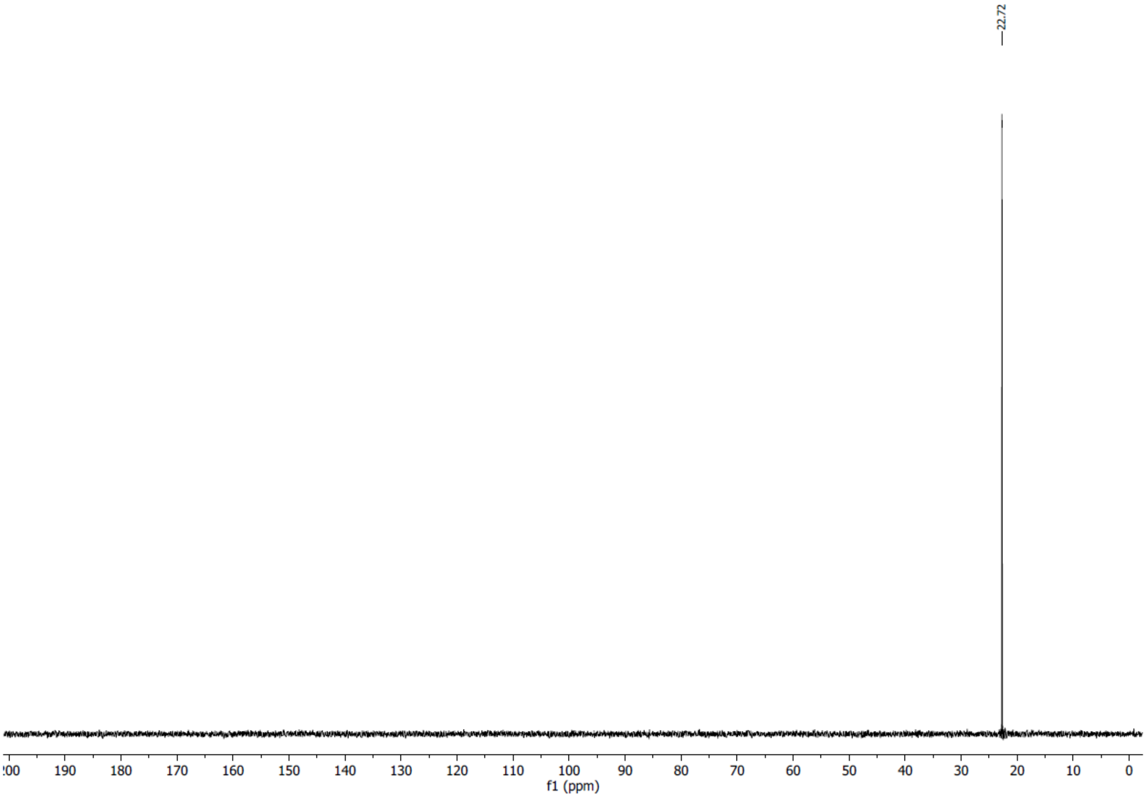

Figure S21. ^{31}P NMR (162 MHz, $CDCl_3$) of 1-(dibenzylphosphoryl)ethyl dodecanoate (**9**).

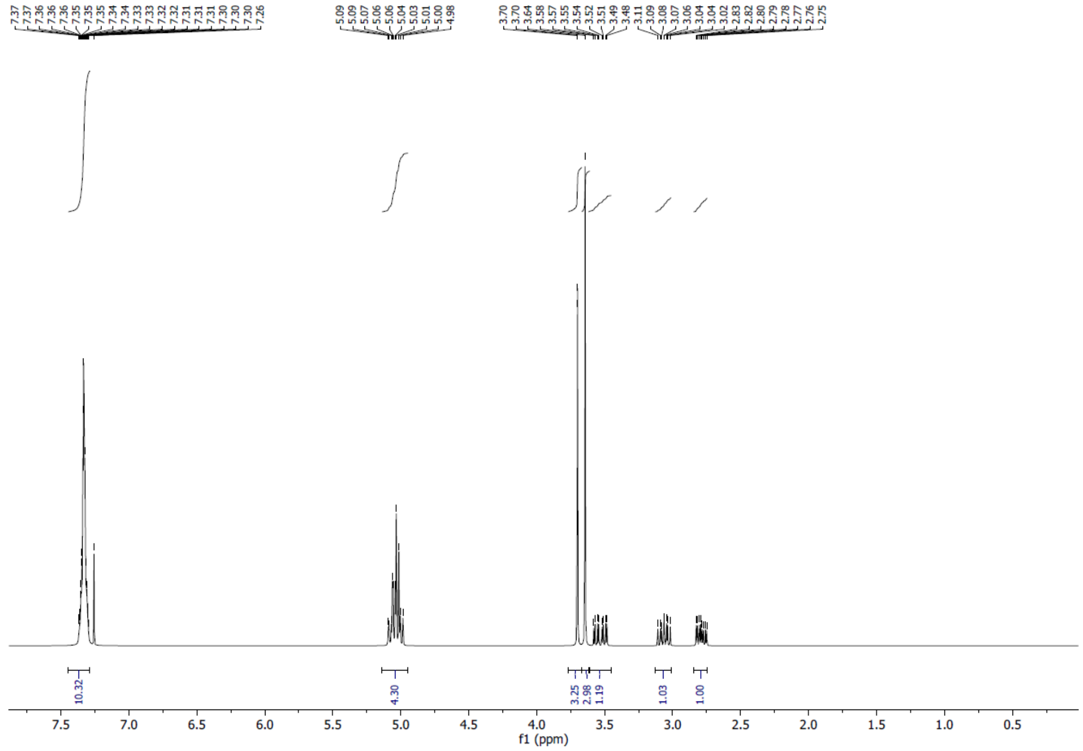

Figure S22. ^{1}H NMR (400 MHz, $CDCl_3$) of dimethyl 2-(dibenzyloxyphosphoryl)succinate (**10**).

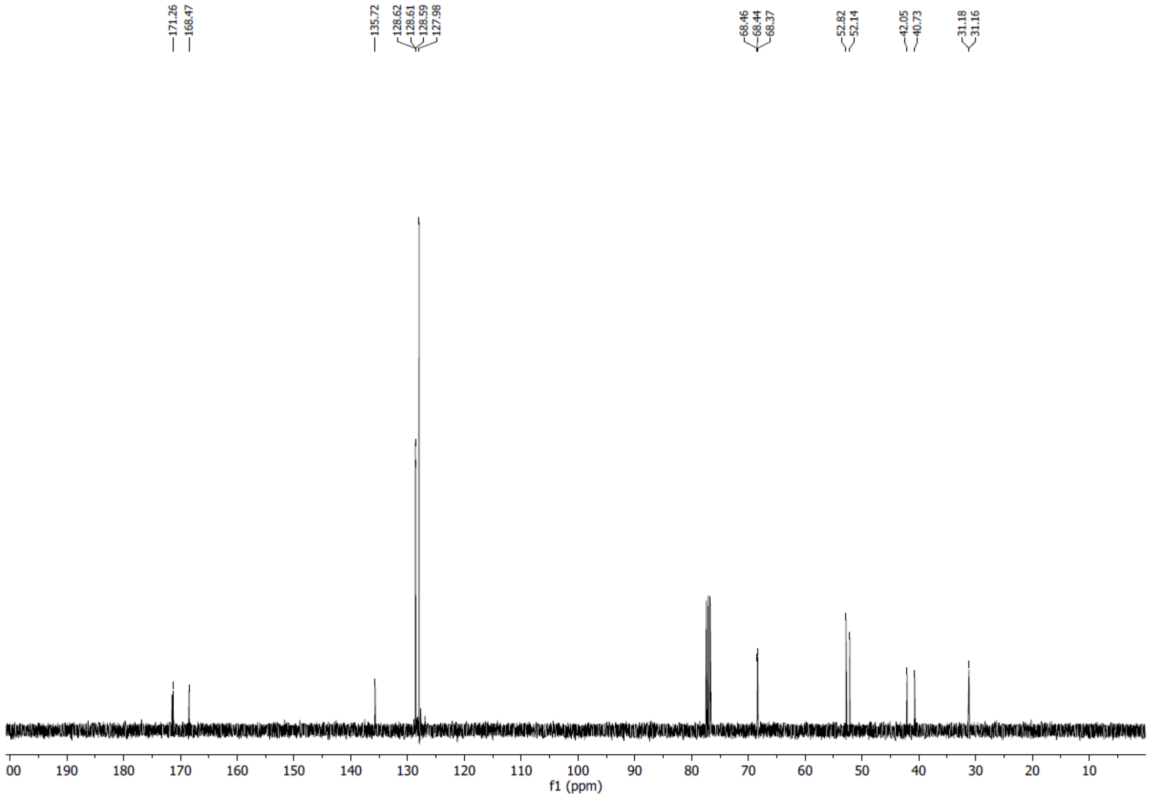

Figure S23. ^{13}C NMR (100 MHz, $CDCl_3$) of dimethyl 2-(dibenzyloxyphosphoryl)succinate (**10**).

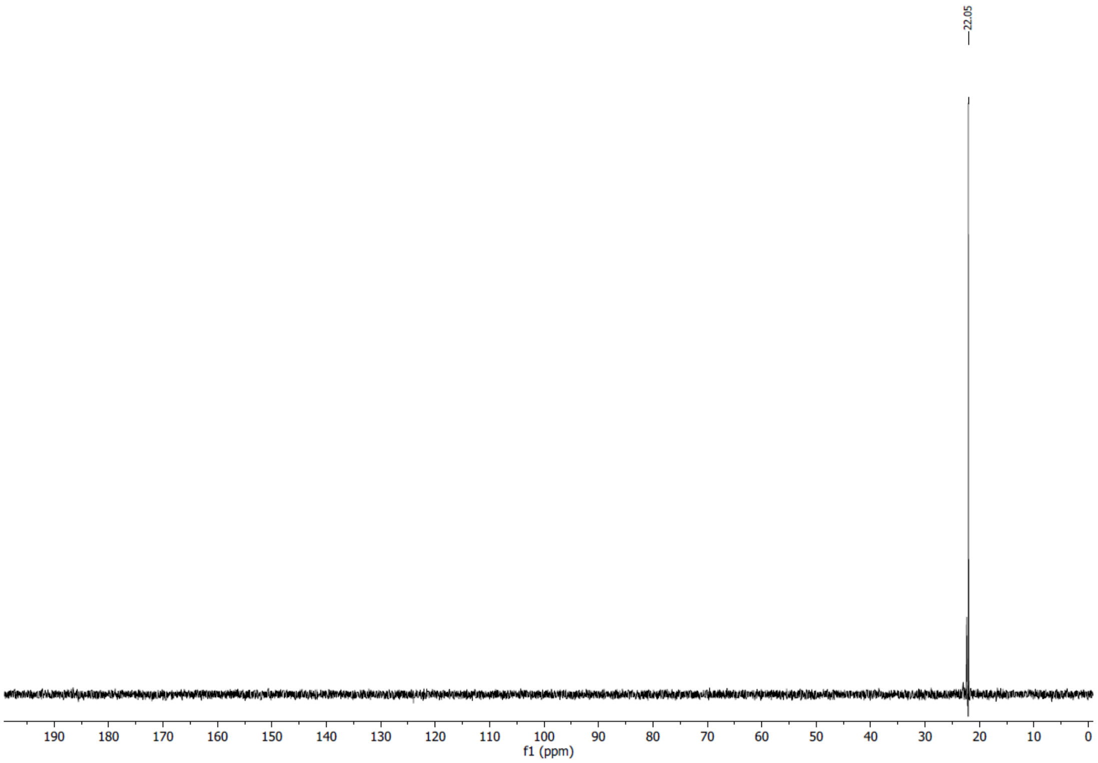

Figure S24. ^{31}P NMR (162 MHz, $CDCl_3$) of dimethyl 2-(dibenzyloxyphosphoryl)succinate (**10**).
